# Blinking characteristics analyzed by a deep learning model and the relationship with tear film stability in children with long-term use of orthokeratology

**DOI:** 10.3389/fcell.2024.1517240

**Published:** 2025-01-28

**Authors:** Yue Wu, Siyuan Wu, Yinghai Yu, Xiaojun Hu, Ting Zhao, Yan Jiang, Bilian Ke

**Affiliations:** ^1^ Department of Ophthalmology, Renji Hospital, Shanghai Jiao Tong University School of Medicine, Shanghai, China; ^2^ Department of Ophthalmology, Shanghai General Hospital, Shanghai Jiao Tong University School of Medicine, National Clinical Research Center for Eye Diseases, Shanghai, China; ^3^ School of Health Science and Engineering, University of Shanghai for Science and Technology, Shanghai, China

**Keywords:** orthokeratology, children, blinking pattern, tear film, deep learning system

## Abstract

**Purpose:**

Using deep learning model to observe the blinking characteristics and evaluate the changes and their correlation with tear film characteristics in children with long-term use of orthokeratology (ortho-K).

**Methods:**

31 children (58 eyes) who had used ortho-K for more than 1 year and 31 age and gender-matched controls were selected for follow-up in our ophthalmology clinic from 2021/09 to 2023/10 in this retrospective case-control study. Both groups underwent comprehensive ophthalmological examinations, including Ocular Surface Disease Index (OSDI) scoring, Keratograph 5M, and LipiView. A deep learning system based on U-Net and Swim-Transformer was proposed for the observation of blinking characteristics. The frequency of incomplete blinks (IB), complete blinks (CB) and incomplete blinking rate (IBR) within 20 s, as well as the duration of the closing, closed, and opening phases in the blink wave were calculated by our deep learning system. Relative IPH% was proposed and defined as the ratio of the mean of IPH% within 20 s to the maximum value of IPH% to indicate the extent of incomplete blinking. Furthermore, the accuracy, precision, sensitivity, specificity, F1 score of the overall U-Net-Swin-Transformer model, and its consistency with built-in algorithm were evaluated as well. Independent t-test and Mann-Whitney test was used to analyze the blinking patterns and tear film characteristics between the long-term ortho-K wearer group and the control group. Spearman’s rank correlation was used to analyze the relationship between blinking patterns and tear film stability.

**Results:**

Our deep learning system demonstrated high performance (accuracy = 98.13%, precision = 96.46%, sensitivity = 98.10%, specificity = 98.10%, F1 score = 0.9727) in the observation of blinking patterns. The OSDI scores, conjunctival redness, lipid layer thickness (LLT), and tear meniscus height did not change significantly between two groups. Notably, the ortho-K group exhibited shorter first (11.75 ± 7.42 s vs. 14.87 ± 7.93 s, p = 0.030) and average non-invasive tear break-up times (NIBUT) (13.67 ± 7.0 s vs. 16.60 ± 7.24 s, p = 0.029) compared to the control group. They demonstrated a higher IB (4.26 ± 2.98 vs. 2.36 ± 2.55, p < 0.001), IBR (0.81 ± 0.28 vs. 0.46 ± 0.39, p < 0.001), relative IPH% (0.3229 ± 0.1539 vs. 0.2233 ± 0.1960, p = 0.004) and prolonged eye-closing phase (0.18 ± 0.08 s vs. 0.15 ± 0.07 s, p = 0.032) and opening phase (0.35 ± 0.12 s vs. 0.28 ± 0.14 s, p = 0.015) compared to controls. In addition, Spearman’s correlation analysis revealed a negative correlation between incomplete blinks and NIBUT (for first-NIBUT, r = −0.292, p = 0.004; for avg-NIBUT, r = −0.3512, p < 0.001) in children with long-term use of ortho-K.

**Conclusion:**

The deep learning system based on U-net and Swim-Transformer achieved optimal performance in the observation of blinking characteristics. Children with long-term use of ortho-K presented an increase in the frequency and rate of incomplete blinks and prolonged eye closing phase and opening phase. The increased frequency of incomplete blinks was associated with decreased tear film stability, indicating the importance of monitoring children’s blinking patterns as well as tear film status in clinical follow-up.

## 1 Introduction

Myopia is a common refractive error that causes distant objects to appear blurry. The global prevalence of myopia is rapidly increasing, making it a significant public health concern (Dong et al., 2020; Holden et al., 2016). Orthokeratology (ortho-K) have emerged as one of the most effective non-surgical methods for myopia control (Bullimore and Johnson, 2020; VanderVeen et al., 2019), offering a practical solution to reduce refractive errors (Vincent et al., 2021; Sánchez-García et al., 2021). However, the growing use of ortho-K has raised concerns about their potential impact on the ocular surface, particularly regarding the risk of inducing dry eye in children (Tao et al., 2021; Sartor et al., 2024). Ortho-K apply direct pressure to the corneal surface overnight, which may alter the distribution, composition, and volume of the tear film (Yang L. et al., 2021). Children with prolonged ortho-K use often exhibit tear film instability and symptoms of contact lens-related dry eye. (Xie and Wei, 2023; Hui et al., 2022; Hou et al., 2023; Li et al., 2023).

Several studies have reported that the instability of the tear film is often accompanied by changes in blink pattern in various ocular surface diseases, particularly dry eye disease (DED) (Rodriguez et al., 2018; Zheng et al., 2022; Jie et al., 2019; Brahmbhatt et al., 2023). Su et al. found that DED patients exhibit a longer eyelid closed time compared to healthy controls (Su et al., 2018). A blink is defined as rapid eyelid closing and opening movements, and normal blink rate is about 12 blinks/minute (Rodriguez et al., 2018). Regular and complete blinking is crucial for maintaining tear film integrity, as incomplete blinking can lead to inadequate lipid distribution and contribute to tear film instability (Jie et al., 2019; Hirota et al., 2013; Wang et al., 2018). However, traditional blink parameters (blink rate, incomplete blink rate) fail to capture the continuous and dynamic nature of the blinking process. Analyzing each phase of the blink process could provide valuable insights into the relationship between ocular health and blinking behavior. Currently, the comprehensive blinking patterns of children with long-term use of ortho-K remain unknown.

In recent years, deep learning has achieved significant advancements in ophthalmology, with high sensitivity and specificity (Sonmez et al., 2024). A deep learning model has been trained and validated to analyze blink videos recorded by Keratograph 5M(15), but the discrepancy in frame rate and capturing light limited the direct transferability of the model to LipiView, a common clinical device for ocular assessment (Li et al., 2020; Chen et al., 2017). Additionally, it is unable to capture detailed blink phase characteristics, further limiting its utility in analyzing complex blinking patterns. To address these gaps, we aimed to develop a deep learning system that can accurately assess blink characteristics including incomplete blink and duration of each blink phase. Subsequently, we will evaluate the changes in blinking patterns and their correlation with tear film characteristics in children with long-term use of ortho-K.

## 2 Methods

### 2.1 Participants

The study protocol was approved by the ethics committee of Shanghai General Hospital, Shanghai Jiao Tong University (approval number [2021KY019]) in accordance with the Declaration of Helsinki. Informed consents were obtained from all the participants and their guardians. Children who had long-term follow-up at optometry clinic of Shanghai General Hospital were recruited. Inclusion criteria for this study were as follows: (1) Aged 6–18 years old; (2) Spherical equivalence (SE) between −0.5D and −5.0D; (3) For the long-term ortho-K used group, continuous usage of spherical four-zone designed ortho-K (Euclid Systems Corporation, Herndon, USA) for more than 1 year and (4) Ability to cooperate with examinations independently. Exclusion criteria included: (1) Current ocular inflammation; (2) Usage of eye drops or ointments within the past 3 months; (3) History of ocular surgery; (4) Eyelid anomalies and (5) Any other ophthalmic or systemic diseases that may affect the ocular surface, such as keratoconus or autoimmune diseases. Finally, 31 children (58 eyes) with long-term use of ortho-K (OK group) and 31 age- and gender-matched normal controls (NC group) (58 eyes) were included in this study.

### 2.2 Ocular examinations

All the participants have undergone the preliminary checkup including detailed medical history, slit-lamp examination, visual acuity assessment, and intraocular pressure measurement. The Ocular Surface Disease Index (OSDI) questionnaire was used to comprehensively assess ocular symptoms, vision-related functions, and environmental triggers. The four-point scale questionnaire comprised 11 questions (excluding the night driving item), focusing on issues experienced within the past week, such as photophobia, foreign body sensation, eye discomfort, blurred vision, decreased visual acuity; impact on activities like reading, using a computer, and watching television; as well as discomfort in windy, dry, and air-conditioned environments. Total OSDI score was calculated as the sum of all scores multiplied by 25, divided by the number of questions answered, with higher scores indicating more severe symptoms.

Examinations were conducted by the same ophthalmologist and a 5-min break was set between tests. Ocular surface and tear film stability assessment were conducted through Keratograph 5M (K5M; Oculus Optikgeräte GmbH, Wetzlar, Germany). Tear meniscus height (TMH) at the center of lower eyelid margin, non-invasive tear film break-up time (NIBUT) for the first time (first-NIBUT) and in average (avg-NIBUT), and conjunctival hyperemia index analyzed by the built-in software were conducted in sequence.

The LipiView interferometer (Johnson & Johnson, USA) was used to evaluate lipid layer thickness (LLT) and blinking patterns. Participants were instructed to gaze naturally at a flashing white light during the examination. The focus was adjusted until the reflection of the lower eyelid eyelashes and the tear film image were clearly visible. LLT was quantified based on interferometric color units. At the same time, LipiView captured a 20-s video at 30 frames per second, with a resolution of 800 × 600 pixels. Measurements with a conformance factor below 0.7 were repeated to ensure accuracy. The frequency of total blinks (TB), incomplete blinks (IB), and complete blinks (CB) and incomplete blinking rate (IBR) was recorded, and the blinking video was saved for further analysis.

### 2.3 Architecture of deep learning system

**FIGURE 1:**
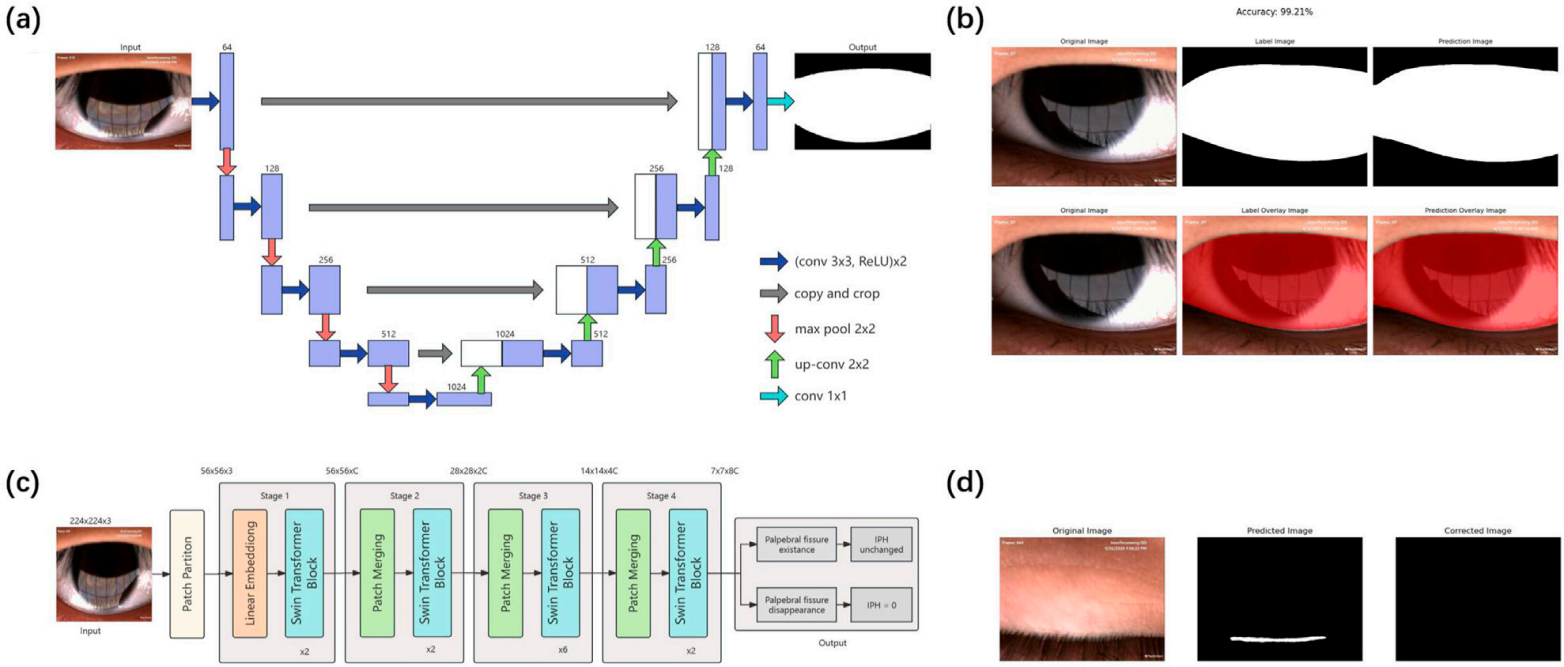
Flow chart of the proposed eyelid region segmentation and blink pattern classification model **(A)** Schematic of the U-Net-based eyelid region segmentation model. The pre-processed single-frame image undergoes four down-sampling and up-sampling steps through the U-Net model, then output the predicted eyelid region segmentation. **(B)** Example outcomes of the eyelid region segmentation model. The left panel shows the original input; the middle panel displays the manually annotated segmentation, and the right panel present the model’s predicted result. **(C)** Schematic of the Swin-Transformer-based blink pattern classification model. The input images were classified into either palpebral fissure existence or palpebral fissure disappearance after passing through four stages of the Swin-Transformer. Palpebral fissure disappearance classifications lead to IPH correction to zero, while palpebral fissure existence classifications remain unchanged. **(D)** Example of classification model correction. The left panel shows an original palpebral fissure disappearance image erroneously segmented as palpebral fissure existence (middle panel). The classification model correctly identifies the palpebral fissure disappearance with 91% confidence, adjusts the segmentation, and sets the IPH to zero (right panel). IPH, interpalpebral height.

Our study employed a deep learning system to analyze blinking patterns and phases in these children. The system comprises two components: the eyelid region segmentation model and the blink pattern classification model (Figure 1).

#### 2.3.1 Data preparation

Blinking videos recorded at 30 frames per second were employed to extract individual frames. Two trained and experienced junior ophthalmologists (WY and WSY) independently annotated the regions between the upper and lower eyelid margins using the Labelme tool and reviewed each other’s annotations. In cases of disagreement, a third senior ophthalmologist (JY) provided the final determination. The interpalpebral height (IPH), defined as the maximum vertical distance between these margins in each frame, was calculated based on the segmentation results. Meanwhile, each frame was classified as either palpebral fissure existence or palpebral fissure disappearance, which was used as the label for training the blink pattern classification model. A total of 1,440 images were collected and divided into training, validation, and test sets in an 8:1:1 ratio. To enhance the model’s generalization ability, a series of data preprocessing operations were applied to the training set. For the eyelid region segmentation model, these operations included: (1) resizing the images and annotations to 512 × 512 pixels by using the nearest-neighbor interpolation, (2), performing random horizontal and vertical flips, and (3) converting the original images into grayscale images. For the blink pattern classification model, the operations included: (1) randomly cropping the images and resizing them to 224 × 224 pixels, (2), randomly flipping them horizontally, and (3) normalizing them using specific mean and standard deviation values. Moreover, to assess interobserver consistency in eyelid region segmentation and IPH measurements, 30 images were randomly selected and independently annotated by two experienced ophthalmologists. The Kappa coefficient of IPH was calculated, resulting in a value of 0.83, indicating high interobserver reliability.

#### 2.3.2 Eyelid region segmentation model

The eyelid region segmentation model utilized a U-Net convolutional neural network. The U-Net model’s network structure can be divided into two main parts: downsampling and upsampling. The left half, the encoder, consists of multiple repetitions of two 3 × 3 convolutional layers followed by a 2 × 2 max-pooling layer. With each downsampling step, the number of channels doubles. The right half, the decoder, is composed of multiple repetitions of a 2 × 2 upsampling layer followed by two 3 × 3 convolutional layers. The feature maps obtained from each upsampling step are concatenated with the corresponding feature maps from the downsampling path. Finally, a 1 × 1 convolutional layer is used to classify each pixel, producing the segmentation result of the input image. The model was trained to segment the closed regions formed by the upper and lower eyelid margins, defining the eyelid region.

To train the U-Net model, the Dice Similarity Coefficient (DSC) was employed as the loss function, and the Stochastic Gradient Descent (SGD) algorithm was used as the optimizer. The initial learning rate was set to 0.01, with a momentum of 0.9. The learning rate was decreased by a factor of 0.1 every 20 epochs, with training proceeding for 100 epochs and a batch size of 4. Early stopping was implemented if the DSC did not improve for 20 consecutive epochs. The model achieving the highest accuracy on the validation set, 97.70%, was selected for segmenting all frames in the blink videos.

#### 2.3.3 Blink pattern classification model

In addition to segmentation, we developed a blink pattern classification model based on the Swin-Transformer architecture. The input to the Swin-Transformer model is an image with dimensions H×W×3. First, the image is processed by the Patch Partition layer, which divides the image into patches. The partitioned data then passes through the Linear Embedding layer for feature mapping. The feature-mapped data is input into the Swin Transformer blocks, forming Stage 1 together with the Linear Embedding. Unlike Stage 1, Stages 2–4 require Patch Merging for downsampling before inputting into the Swin Transformer blocks. Finally, the output from Stage 4 is used to classify each frame as either palpebral fissure existence or palpebral fissure disappearance. The classification model was trained using the cross-entropy loss function and the AdamW optimizer, with an initial learning rate of 0.0001 and a weight decay parameter of 5E-2. The batch size was set at 8, and the model was trained for 10 epochs. It achieved an accuracy of 99.59% on the validation set.

The blink pattern classification model’s predictions were used to determine the condition of palpebral fissure for each frame, allowing for the verification and correction of the segmentation results. If the classification model identified a frame as palpebral fissure disappearance and the IPH determined by the UNet was non-zero, the segmentation result was corrected to an unannotated background image, with the corrected IPH set to zero. No correction was needed in other cases.

### 2.4 Analysis of the blink wave and phase duration

To analyze blinking patterns, we processed all frames from the blink videos using our eyelid region segmentation and blink pattern classification models. Each frame’s interpalpebral height (IPH) was calculated and normalized by dividing it by the image height, resulting in interpalpebral height percentages (IPH%). These percentages were plotted over time to create blink waves, providing a visual representation of blinking activity over 20 s.

**FIGURE 2:**
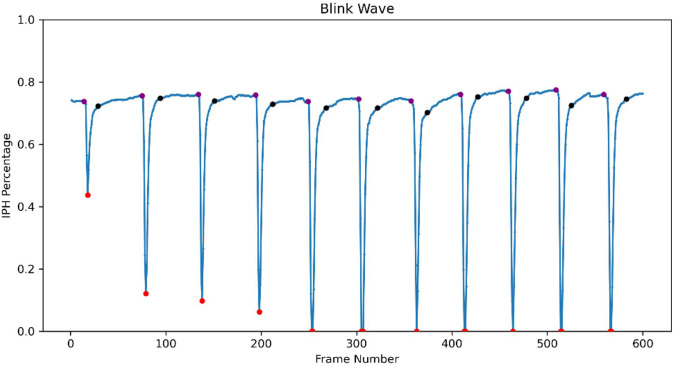
Blink wave generated from the deep learning model of LipiView-recorded blinking video. Interpalpebral height percentages (IPH%) for each frame were analyzed and plotted over time, generating a blink wave. The purple dot indicates the start of the closing phase and its corresponding baseline IPH%. The red dot marks the blink wave trough, representing the end of the closing phase and the start of the opening phase. The black dot represents the end of the opening phase.

We identified the start and end of each blink by analyzing changes in IPH% between frames (Figure 2). A blink was classified as complete if the lowest IPH% during the blink was 0%, and incomplete if it was greater than 0%. If the lowest IPH% exceeded 80% of the baseline, it was not considered a blink. We further defined and calculated relative IPH% as the ratio of the mean of IPH% within 20 s to the maximum value of IPH%. A higher relative IPH% value indicates a greater extent of incomplete blinking. Moreover, each blink was divided into three phases: (1) Closing Phase: From the beginning of eyelid closure to full closure; (2) Closed Phase: When the eyelids are fully closed (absent in incomplete blinks); (3) Opening Phase: From full closure back to the open-eye height. We define ΔIPH% as the difference in IPH% between two successive frames. If five consecutive ΔIPH% >0, the first of these six frames marks the blink start. Conversely, if five consecutive ΔIPH% <0, the last of these six frames marks the blink end. We quantified the frequency of incomplete blinks, complete blinks, and total blinks in each video. The duration of each phase within a blink was also examined to understand blinking characteristics across groups.

### 2.5 Statistical analysis

SPSS 25.0 statistical software (SPSS Inc., Chicago, IL, United States) was applied for statistical analysis. Data were presented in mean ± standard deviation unless otherwise stated. Shapiro-Wilk test was used to test the normality of parameters. Bland-Altman test and Intraclass correlation coefficient (ICC) of blinking pattern metrics from the LipiView and our system were calculated to evaluate the measurement consistency. Student’s t-test and Mann-Whitney U test was applied to detect the differences of tear stability, blinking pattern and blink wave phase duration between OK group and the control group. Spearman correlation test was used to analyze the relationship between ocular surface parameters and blinking characteristics. P < 0.05 was considered statistically significant.

## 3 Results

### 3.1 Validation of our deep learning system

A total of 116 eyes of 62 participants were included in this study. Demographic and general characteristics of ocular surface are presented in Table 1.

**TABLE 1:** Characteristics of the participants.

Variable	OK group (n = 58, eye)	NC group (n = 58, eye)	P Value
Age, y	12.12 ± 2.29	12.16 ± 2.30	0.936
Gender, male	38	38	1.000
Wearing time, y	2.28 ± 1.45	0	<0.001
OSDI	4.61 ± 4.73	4.56 ± 4.76	0.960
Redness index	0.656 ± 0.40	0.887 ± 0.33	0.221
LLT, nm	60.36 ± 21.22	62.67 ± 24.263	0.593
Meniscus height, mm	0.20 ± 0.05	0.22 ± 0.05	0.079
1st-NIBUT, s	11.75 ± 7.42	14.87 ± 7.93	0.030*
Avg-NIBUT, s	13.67 ± 7.0	16.60 ± 7.24	0.029*

Data are presented as means ± SD. *P < 0.05.

For the U-Net segmentation model, dice similarity coefficient (DSC), Intersection over union (IOU), Balanced accuracy (BAC), and Sensitivity (SEN) was used to validate its performance. High performance metrics (DSC = 0.9594 ± 0.0465, IOU = 0.9255 ± 0.0774, BAC = 0.9726 ± 0.0265, SEN = 0.9617 ± 0.0546) demonstrate the robustness of the model.

As shown in Table 2, the overall accuracy of initial U-Net-based model is 78.17%, and was improved to 98.13% by integrating a Swin-Transformer model, and the sensitivity and the F1 score of the model were also increased from 36.94% to 98.10% and from 0.5353 to 0.9727, respectively. The improved model also demonstrated high levels of precision and specificity (precision = 96.46%, specificity = 98.10%).

**TABLE 2:** Performance of U-Net and U-Net-Swin-Transformer model.

Models	Accuracy	F1 score	Precision	Sensitivity	Specificity
U-Net	0.7817	0.5353	0.9879	0.3694	0.9976
U-Net-Swin-Transformer	0.9813	0.9727	0.9646	0.981	0.981

**FIGURE 3:**
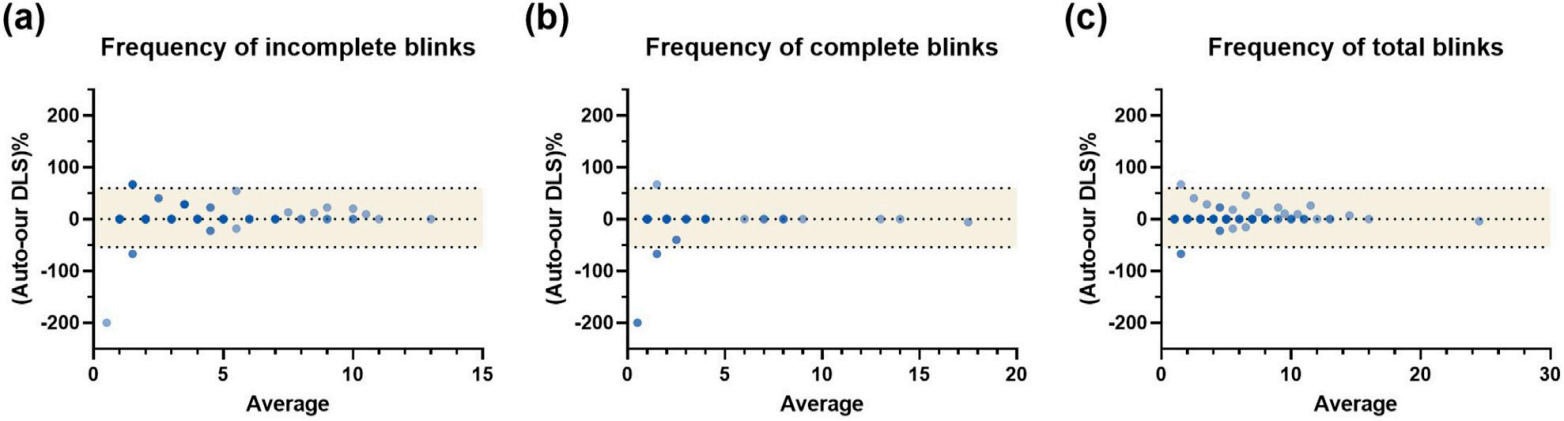
Bland-Altman analysis of the blinking pattern parameters between built-in algorithm and our system **(A)** The Bland-Altman analysis of frequency of IB; **(B)** The Bland-Altman analysis of frequency of CB; **(C)** The Bland-Altman analysis of frequency of TB. An agreement was observed between the built-in LipiView algorithm and the proposed deep learning system for IB, CB, and TB measurements. IB, incomplete blink; CB, complete blink; TB, total blink; DLS, deep learning system.

Bland-Altman analysis was performed to evaluate the consistency between built-in algorithm and our system. For the frequency of incomplete blinks, our system showed a paired difference of 2.81% compared to the built-in system, with the 95% limits of agreement ranging from −54% to 59.6%. Regarding the frequency of complete blinks, the paired difference was −8.24%, with 95% limits of agreement from −81.4% to 64.9%. For the total frequency of blinks, the paired difference was 1.10%, with the 95% limits of agreement ranging from −27.3% to 29.5%. The consistency of measurements was also evaluated in both devices. Our deep learning system demonstrated higher ICC values (0.991, 0.998, 0.995) in these parameters separately (Figure 3).

### 3.2 Tear film instability in children with long-term ortho-K use

The OSDI scores were 4.61 ± 4.73 in the OK group and 4.56 ± 4.76 in the control group, showing no significant difference between the groups (p = 0.960). Neither group exhibited corneal staining, and there was no significant difference in the conjunctival redness index (p = 0.221), indicating that long-term use of ortho-K did not result in a significant adverse impact on the ocular surface, and the overall ocular surface health was comparable between the two groups. The lipid layer thickness (LLT) was 60.36 ± 21.22 nm in the OK group and 62.67 ± 24.26 nm in the control group, with no statistically significant difference (p = 0.593). Similarly, the tear meniscus height did not differ significantly between the two groups (p = 0.079). However, significant differences were found in both first-NIBUT and avg-NIBUT between the two groups. The OK group exhibited significantly shorter tear break-up times compared to the control group (p = 0.030 and p = 0.029, respectively), indicating increased tear film instability in children with long-term use of ortho-K (Table 1).

### 3.3 Changes in blinking characteristics in children with long-term ortho-K use

**FIGURE 4:**
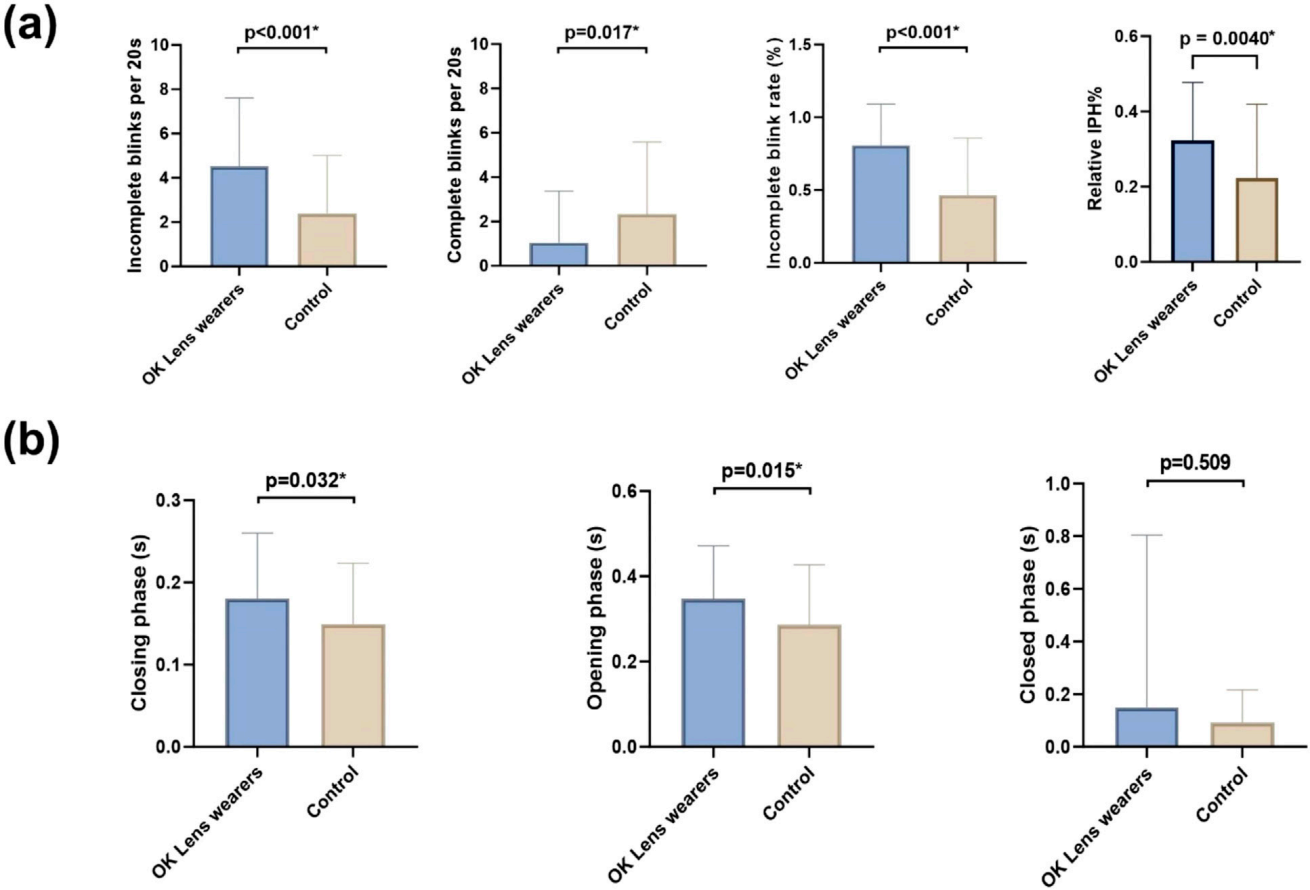
Blinking characteristics difference in the OK group and the control group **(A)**. Comparison of blink characteristics between the two groups including frequencies of IB, CB, IBR, and relative IPH%; **(B)**. Comparison of blink phase durations (closing, closed, and opening phases) between the two groups. Significant differences were observed in blink characteristics and phase durations. IB, incomplete blink; CB, complete blink; IBR, incomplete blink rate; IPH, interpalpebral height. *P < 0.05.

Despite no significant difference in the total frequency of blinks between the two groups (p = 0.170), there were notable differences in the blinking patterns. Children in the OK group exhibited a significantly higher frequency of incomplete blinks within a 20-s period (4.26 ± 2.98) compared to the control group (2.36 ± 2.55) (p < 0.001). Lower frequency of complete blinks was also observed within a 20-s period in the OK group (1.10 ± 2.35) compared to the control group (2.38 ± 3.33) (p = 0.017). The rate of incomplete blinks was significantly higher in the OK group (0.81 ± 0.28) compared to the control group (0.46 ± 0.39) (p < 0.001). Furthermore, the relative IPH% showed significant difference between two groups (0.3229 ± 0.1539 vs 0.2233 ± 0.1960, p = 0.004) (Figure 4A).

Analysis of the three phases of blinking revealed that the OK group had a significantly longer eye-closing phase duration (0.18 ± 0.08 s) compared to the control group (0.15 ± 0.07 s) (p = 0.032). The eye-opening phase duration was significantly higher in the OK group (0.35 ± 0.12 s) when compared to the control group (0.28 ± 0.14 s) (p = 0.015). There were no significant differences in the durations of the closed phase between the two groups (p > 0.05) (Figure 4B).

**FIGURE 5:**
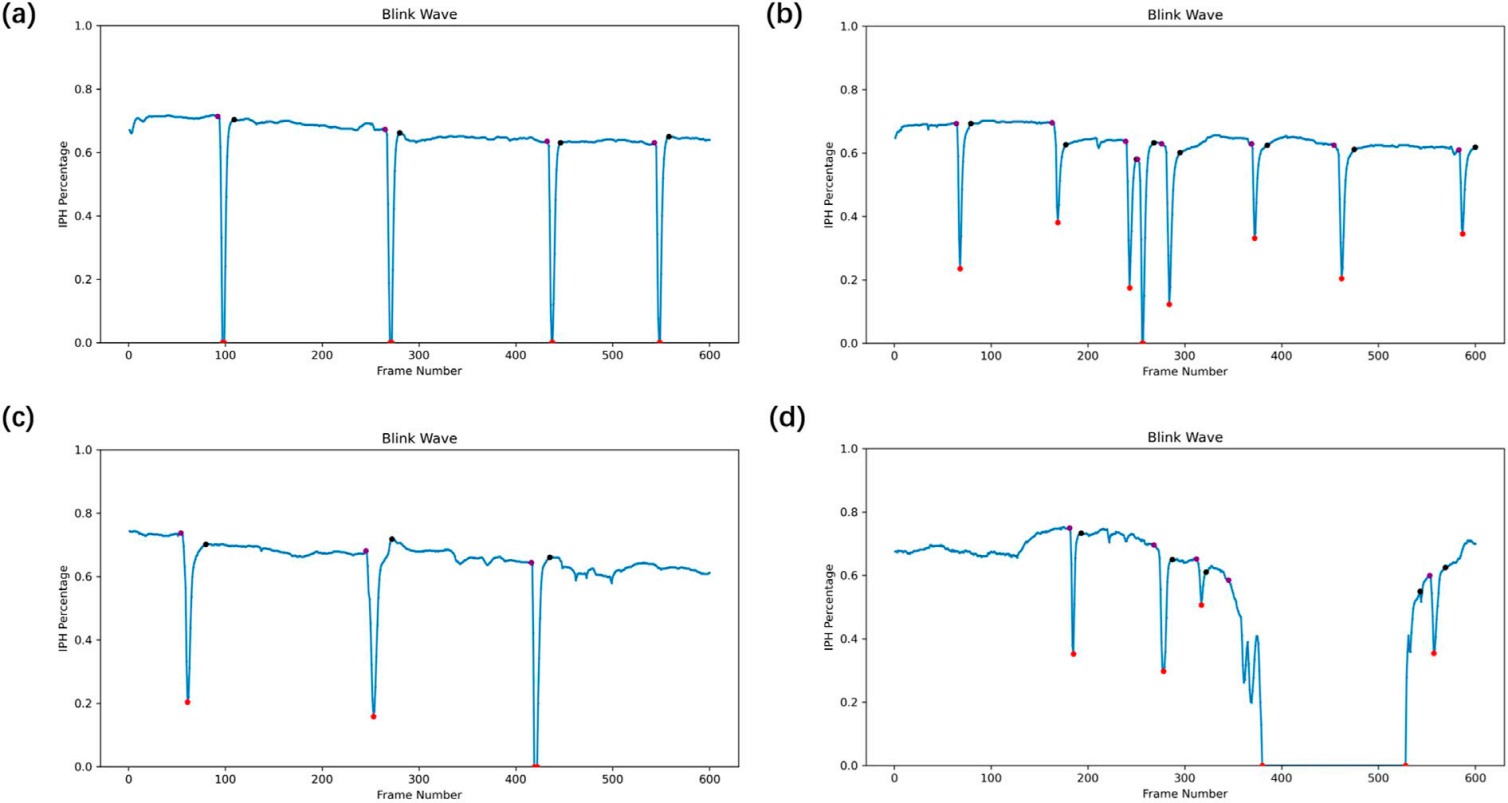
Representative blink waves in the OK group and the control group **(A–D)**. **(A)** Complete blinks in the control group with symmetrical waveforms. **(B)** Incomplete blinks in the OK group exhibit incomplete closure. **(C)** Prolonged eye-closing and eye-opening phases in the OK group suggest altered blink dynamics. **(D)** Abnormally prolonged eye-closed phase was observed in the OK group.

Overall, the blinking patterns of children in the OK group were more irregular and asymmetrical compared to the control group (Figure 5). Incomplete blinks, prolonged eye-closing and opening phase were the three main characteristics of their blink patterns, indicating altered and potentially less efficient blinking patterns.

### 3.4 Correlation between tear stability and blinking characteristic

**FIGURE 6:**
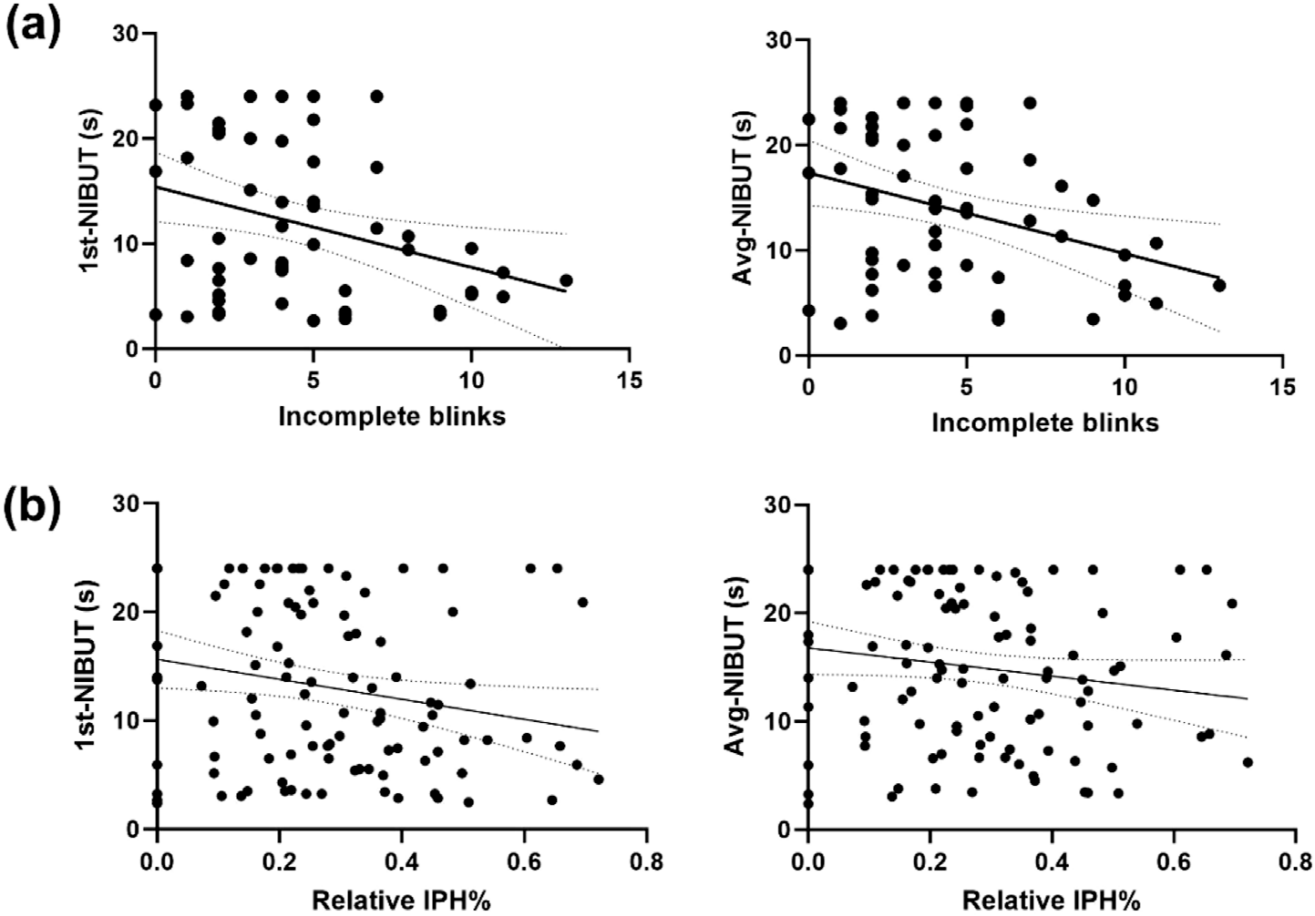
Correlation analysis between blinking patterns and tear film characteristics **(A)**. The frequency of incomplete blinks negatively correlates with first-NIBUT (r = −0.292, p = 0.004) and avg-NIBUT (r = −0.3512, p < 0.001) in the OK group. **(B)** Relative IPH% also negatively correlates with first-NIBUT (r = −0.215, p = 0.026) and avg-NIBUT (r = −0.196, p = 0.042), suggesting a link between incomplete blinking and tear film instability.

In OK group, Spearman’s rank correlation assessment of blinking patterns and tear film quality found the frequency of incomplete blinks had significant negative correlation with NIBUT (first-NIBUT, r = −0.292, p = 0.004; avg-NIBUT, r = −0.3512, p < 0.001) (Figure 6A). Similarly, the value of relative IPH% showed significant negative correlation with NIBUT in all participants (first-NIBUT, r = −0.215, p = 0.026; avg-NIBUT, r = −0.196, p = 0.042) (Figure 6B). However, no significant correlation was observed between LLT (r = −0.221, p = 0.095), meniscus height (r = 0.255, p = 0.055) and incomplete blinks. In addition, we did not observe any significant correlation between NIBUT, LLT, meniscus height and blinking phase.

## 4 Discussion

While orthokeratology are commonly used to manage myopia progression in children, their long-term effects on ocular surface health needs further investigation. In this study, we applied a deep learning model to obtain blinking characteristics comprehensively, aiming to evaluate the impact of long-term ortho-K wear on blinking patterns and tear film stability in children more precisely.

Blinking is a complex and dynamic process that is influenced not only by human consciousness but also by the condition of the ocular surface. Previous studies have attempted to describe and explore this process in patients with dry eye disease (DED). Ousler et al. (Ousler et al., 2014) analyzed spontaneous blink videos of 21 participants and found that DED patients have longer eye-closed time. Su et al. (Su et al., 2018)analyzed spontaneous blink videos of 50 participants and found a significant increase in frequency of incomplete blinks among DED patients compared to controls. However, both studies used manual blink video analysis, which, despite its reliability, is labor-intensive and time-consuming, limiting its effectiveness and potentially impacting the validity of their results.

Deep learning has proven its strength dealing with large-scale data in classification and segmentation tasks. Zheng et al. (2023) introduced a texture fusion segmentation network (TF-Net) by incorporating two different convolutional blocks into the U-Net architecture to extract the palpebral fissure from ocular images. Their network achieved an average Dice score (DS) of 0.9445 on a blink image dataset. However, their deep learning model only analyzed the total frequency of blinks in each video, without addressing the different stages of each blink. Our research employed a combined UNet (Fischer et al., 2015) and Swin-Transformer (Liu et al., 2021) model for blink characteristic analysis. This approach allowed us to effectively capture blink waves and analyze both blink frequency and the duration of each blink phase. Our model achieved a Dice Similarity Coefficient (DSC) of 95.94% on the test set, while the blink pattern classification model attained an accuracy of 99.59% on the validation set.

Previous studies have indicated that short-term overnight use of ortho-K for 1 week increase the incomplete blinks rate (Zeng et al., 2020), but the long-term effects on children’s blinking patterns remain uncertain. Our study revealed that children in the long-term ortho-K wearing group had a higher frequency and rate of incomplete blinks compared to the control group. And with the help of the deep learning model, we further defined and observed a higher relative IPH% value in the long-term ortho-K wearing group, indicating that this group exhibited a greater extent of the incomplete blinks and resulting in a smaller area of tear film renewal. A higher incomplete blinks rate might represent an adaptive response that promotes tear film renewal in the central optical zone (Su et al., 2018). Harrison et al. (2008) found that incomplete blinks cause less stretching on the fragile tear film in dry eye patients compared to complete blinks, helping maintain tear film stability. Moreover, previous studies indicated that the eye-closing phase lasts from 0.07s to 0.214s and the eye-opening phase ranges from 0.169s to 0.438s (Choi et al., 2007; Bologna et al., 2009; Kwon et al., 2013). Interestingly, our study found prolonged eye-opening phase as a blink characteristic in children with long-term use of ortho-K. One possible explanation is that long-term manipulation of the upper eyelid during the application and removal of lenses, combined with friction during wear, may potentially affect the levator palpebrae superioris muscle (van den Bosch and Lemij, 1992). Navascues-Cornago et al. (2022) have found that long-term wearers of rigid contact lenses often experience a reduction in palpebral fissure height, and some of them developed acquired blepharoptosis, which is associated with slower eye-opening speeds (Mak et al., 2016; Joo et al., 2021). Further studies are needed to investigate structural and functional changes in the upper eyelid. Moreover, ortho-K may also affect tear film distribution and stability. Uneven tear film distribution may cause ocular discomfort, requiring extended blink durations to redistribute the tear film, which potentially prolong the eye-opening phase. Compared to healthy individuals, Su et al. (2018) found that the duration of early opening phase were significantly longer in dry eye patients, whose tear film were unstable. We found similar results in this study, the prolonged eye-opening phase might enhance tear film coverage, serving as a protective response to restore ocular surface stability.

Due to the low repeatability of questionnaire among children, assessing dry eye symptoms is always challenging (Chidi-Egboka et al., 2021), which makes it more complicate to observe and analyze the impact of ortho-K on the ocular surface. Through a combination of OSDI scores, corneal staining and redness analysis, we observed no significant differences between groups, suggesting a relatively stable ocular surface. Additionally, the overnight use of ortho-K might cause the instability of the tear film, through the direct mechanical friction exerted on the ocular surface (Li et al., 2023; Na et al., 2016), the isolation of the cornea and external air (Panaser and Tighe, 2014) and the corneal reshaping (Li et al., 2018). We therefore compared tear film quality between the two groups and found that the first and average Non-Invasive Break-Up Time (first-NIBUT and Avg-NIBUT) were significantly shorter in the long-term ortho-K wearing group, consistent with previous studies (Tao et al., 2021; Zeng et al., 2020; Na et al., 2016; Li et al., 2018). However, we did not observe significant change in lipid layer thickness (LLT) and tear meniscus height, suggesting that regular long-term use of ortho-K has relatively minimal impact on the composition and volume of tear film. The effect of long-term use of ortho-K on LLT remains unclear. Yu et al. (Yu et al., 2022) found that wearing ortho-K for two or more years did not change the LLT. They proposed a reason that the upper meibomian gland could produce a stable tear film alone after an incomplete blink, thus maintaining the level of LLT. While a Korean study (Lee et al., 2022) reported that wearing ortho-K for at least 4 months was associated with increased LLT and higher meiboscores of lower eyelids, which could be a compensatory mechanism, for the obstruction of the lower eyelid orifice might result in increased lipid secretion in the upper eyelid for the maintenance of homeostasis.

Further analysis indicated a significant negative correlation between NIBUT and incomplete blinks, while no significant association was observed with the blinking phase, revealing that the completion of the blinking process may have a more significant impact on tear film stability than the duration of each phase. In addition, previous studies have demonstrated a mutual relationship between blinking patterns and tear film stability. On the one hand, Yang et al. (Yang B. et al., 2021) revealed that abnormal blinking patterns have been linked to shorter TBUT and decreased LLT in children with allergic conjunctivitis. Jie et al. (2019) found similar results in dry eye patients. Yu et al. (2022) reported that the incomplete blink rate has a significant positive correlation with LLT in long-term ortho-K wearing group. On the other hand, studies (Schlote et al., 2004; Collins et al., 2006) have reported that the condition of the tear film can directly influence the blinking patterns as well. Su et al. (2018) referred that abnormal tear films may trigger incomplete blinks, as a compensatory response. Such unhealthy mutual relationship could result in a vicious cycle, potentially increasing the risk of ocular surface diseases. Wang et al. (2018) found that incomplete blinks was associated with a two-fold increased risk of dry eye disease. Therefore, monitoring blinking patterns and the tear film stability should be emphasized in long-term ortho-K management, in order to prevent ortho-K lens-related dry eye and other ocular surface diseases.

There are several limitations in this study. First, the sample size of our study is relatively small. Nevertheless, *post hoc* power analysis with G*Power software (Faul et al., 2007) based on the results of the frequency of incomplete blinks and our sample size, which indicated a 94.6% detection power in this study. Therefore, it is still sufficient to determine the differences of blinking patterns in children with long-term use of ortho-K. Second, we did not evaluate the morphology and function of the meibomian glands, which could potentially be affected by long-term ortho-K wear (Yu et al., 2022; Lee et al., 2022). Third, the frame rate of LipiView was relatively insufficient to fully capture the details of rapid eye movements, limiting our ability to conduct a more detailed phase classification or accurately assess blink speed. Future studies employed cameras with higher frame rates may provide more precise assessment into these rapid movements.

## 5 Conclusion

In this study, we employed a deep learning model to facilitate the observation of blinking patterns in children with long-term use of ortho-K. Our proposed model achieved optimal performance with increased accuracy and sensitivity. Incomplete blinks, prolonged eye-closing and opening phase were the main characteristics of their blink patterns compared to the healthy control. More incomplete blinks were associated with reduced NIBUT, suggesting that less effective blinking may contribute to tear film stability. Clinicians might need to pay more attention on guiding proper blinking and monitoring tear film health during long-term use of ortho-K in children.

## Data Availability

The datasets presented in this article are not readily available because their containing information that could compromise the privacy of research participants. Requests to access the datasets should be directed to kebilian@126.com.
